# Implementing Adult Hepatitis B Immunization and Screening Using Electronic Health Records: A Practical Guide

**DOI:** 10.3390/vaccines12050536

**Published:** 2024-05-14

**Authors:** H. Nina Kim, Kelly L. Moore, David L. Sanders, Michaela Jackson, Chari Cohen, Richard Andrews, Camilla S. Graham

**Affiliations:** 1 Division of Allergy & Infectious Diseases, Department of Medicine, University of Washington, Seattle, WA 98104, USA; 2 Immunize.org, St. Paul, MN 55116, USA; 3 Department of Health Policy, Vanderbilt School of Medicine, Nashville, TN 37232, USA; 4 Hepatitis B Foundation, Doylestown, PA 18902, USA; 5 Houston Viral Hepatitis Task Force, Houston, TX 77040, USA; 6 Advisory Panel, Hep B United, Doylestown, PA 18902, USA; 7 Division of Infectious Diseases, Beth Israel Deaconess Medical Center and Harvard Medical School, Boston, MA 02215, USA

**Keywords:** hepatitis B, electronic health record, public health, CDC recommendations

## Abstract

Importance: Hepatitis B is a serious problem in the United States (US), with up to 2.4 million Americans living with a chronic infection. Only 26–32% of people living with hepatitis B in the US are diagnosed. Additionally, just 30% of all adults are vaccinated against the virus. In 2022, the Advisory Committee on Immunization Practices of the Centers for Disease Control and Prevention (CDC) updated adult hepatitis B vaccination recommendations to include all adults aged 19–59 years and those 60 years and older with risk factors for hepatitis B. Subsequently, in 2023, the CDC recommended that all adults be screened at least one time in their lives. Observations: Electronic health record (EHR) tools (prompts, order sets, etc.) have proven to be an effective method of increasing hepatitis B screening and vaccination, but longstanding challenges and questions around hepatitis B vaccines and tests could prevent effectual EHR implementation. As the new recommendations directly impact providers who may have limited familiarity with hepatitis B, guidance on how to identify eligible patients and triggers, order sets to facilitate vaccine/test selection, and proper documentation and patient follow-up is necessary. Conclusions and Relevance: This communication offers a practical framework for health systems to build an effective EHR strategy for the updated adult hepatitis B recommendations. We also provide comprehensive responses to clinicians’ questions that are frequently asked prior to screening or vaccinating for hepatitis B.

## 1. Introduction

Chronic hepatitis B (HBV) remains a leading cause of decompensated liver disease and liver cancer worldwide [1]. In the United States, an estimated 2.4 million individuals are living with chronic HBV, but only 26–32% have been diagnosed [2,3]. Only 30% of the adult US population has completed vaccination against HBV infection [4]. This low vaccination rate, along with the ongoing opioid epidemic, has contributed to a rise in acute HBV infections in the US in recent years [5]. In April 2022, the Advisory Committee on Immunization Practices (ACIP) expanded the age range for universal HBV vaccination to include those aged 19–59 years and removed the risk factor assessment previously necessary to determine vaccine eligibility [5]. This recommendation builds on the highly successful universal HBV vaccination program in infants with adolescent “catch up” starting in the 1990s and serves as a catch-up program for adults in the United States. In March 2023, the Centers for Diseases Control and Prevention (CDC) also updated their 2008 HBV screening recommendations to endorse one-time universal HBV screening of all adults in the United States [6].

Electronic point-of-care reminders or prompts have been shown to be an effective strategy for improving delivery of vaccination and other preventive care measures [7,8,9]. Increasingly, health systems are providing decision support and including “choice architecture” in electronic order entry to facilitate best clinical practices [10]. Such electronic health record (EHR)-based strategies have been shown to be effective in increasing rates of HBV screening and vaccination [8].

The goal of this paper is to provide a roadmap for implementing such changes via the EHR to expand HBV vaccination and HBV screening. Our panel of authors includes individuals with expertise in health policy, clinical informatics, and clinical care related to HBV. While we do not provide the actual programming code, we provide the logic underpinning such code so that this can be adapted to multiple EHR platforms and accessible to different disciplines. Any change comes with roadblocks—hepatitis B elimination progress has been challenged by the variety of vaccines, the implications for specific subpopulations, and the multiple options for HBV testing combinations. We provide practical guidance to address questions or concerns that may arise, in alignment with current federal recommendations.

## 2. Summary of HBV Vaccination Recommendations

In April 2022, the CDC published updated recommendations for hepatitis B vaccination from the ACIP [5]. In addition to infant and childhood vaccination, all adults aged 19–59 years should receive an HBV vaccine series if they do not have a history of prior vaccination or of past or current HBV infection. In other words, the decision to vaccinate should be prompted by this age range, regardless of the presence or absence of HBV-related risk factors.

Adults aged 60 years and older who have risk factors for HBV acquisition (Table 1) should also be routinely vaccinated against HBV if they do not have a history of prior vaccination or evidence of past or current HBV infection. Those 60 years of age and older without known risk factors may be offered HBV vaccination if they do not have a history of prior vaccination or evidence of past or current HBV infection. In other words, people with risk factors who are 60 years and older *should* be vaccinated, and people older than 60 years without such factors *may* be vaccinated [5]. In contrast to prior CDC guidelines, the current recommendation places the responsibility on providers (rather than patients) to initiate the discussion of HBV vaccination with all older adult patients.

## 3. EHR Implementation for Vaccination

Implementation of HBV vaccination clinical decision support can be divided into three distinct modules: (1) identification of eligible patients and triggers, (2) order sets to facilitate vaccine selection, and (3) documentation and patient follow-up. Individual EHR capabilities will determine which modules can be built and the complexity of each.

### 3.1. Identification of Eligible Patients and Triggers

The objective is to identify patients eligible for vaccination as accurately as possible using patient-specific data available to the EHR. This may involve simple and complex logic depending on the types of data elements available and the programming capabilities available in the system.

**Figure 1:**
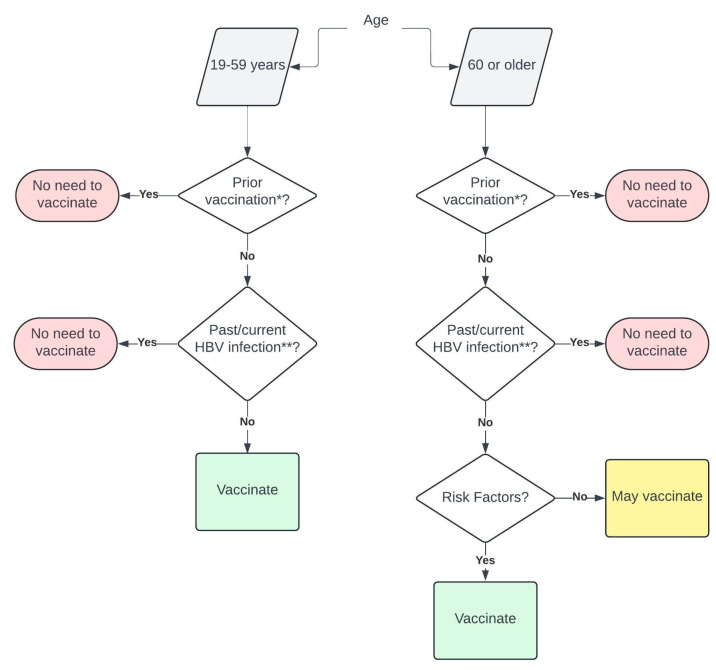
Workflow to assess hepatitis B vaccine eligibility. * Evidence of prior receipt of complete vaccination series requires documentation of the vaccine series or the presence of anti-HB surface antibodies (anti-HBs, HBsAb). ** Serological evidence of past or current HBV infection includes the presence of one or more of the following: HBsAg, anti-HB surface (anti-HBs, HBsAb), anti-HB core total (HBcAb total), HBe antigen (HBeAg), anti-HBe (HBeAb), or detected HBV DNA.

Ideally, the screening logic would identify only patients who are potentially eligible for HBV vaccination using the appropriate inclusion and exclusion criteria. In the case of HBV, this logic would center around age or risk factors (if 60 years of age or older, Table 1) and exclude those who have an active or prior HBV infection or evidence of prior vaccination. Accurate selection of patients eligible for vaccination should maintain high sensitivity while minimizing false-positive alerts to avoid alert fatigue or distrust. The end result will be the classification of each patient as potentially eligible for vaccination or not eligible (Figure 1).

Sources of patient data will depend on EHR capabilities and could include patient demographics, the active problem list, past medical history, list of prior diagnosis code from billing data, HBV test results, and social history.

Many risk factors are not well documented in EHRs or are difficult to extract for prompt development. Institutions may decide to offer universal hepatitis B vaccination to all individuals aged 60 years and older to circumvent this issue.

### 3.2. Order Sets

Order sets should present clinicians with clear and concise guidance while presenting all available vaccine options that may be appropriate for the patient. Vaccines should be preferentially offered as a series to ensure the order stays active until all doses are administered and to avoid the need to re-enter separate doses.

These should include available vaccine choices (see Table 2) and reasons for selecting one vaccine over another (e.g., pregnancy, ESRD/hemodialysis, immunocompromised status). No single vaccine brand or series is appropriate for all patients, but a default brand may be pre-selected (based on efficacy and safety data, the profile of the patient population being served, and formulary placement). Additional information provided about specific patient characteristics can lead to the more appropriate selection of a different brand.

### 3.3. Documentation and Patient Follow-Up

Chart documentation of indication for vaccination should be a part of this workflow, as should documentation of vaccine brand and dose selected and scheduling of follow-up visits and orders.

### 3.4. Guidance for Specific Scenarios (Discussed in the “Frequently Asked Questions” Section)

- HBV screening and administering first vaccine dose on the same day;
- HBV screening in pre-visit orders; decide on vaccination at visit based on results;
- Vaccination without HBV screening.

## 4. Hepatitis B Vaccine Considerations

Four monovalent hepatitis B vaccines are FDA-approved for use in adults in the US (Table 2). Most vaccines have three-dose schedules, and one vaccine is available with a two-dose schedule. Two brands are recommended for use during pregnancy, while two brands are not recommended due to a lack of sufficient safety data on their use during pregnancy. Specific formulations are recommended for individuals on hemodialysis. The dosing schedule of Heplisav-B is two doses given 4 weeks apart. Three monovalent HBV vaccine products (Engerix-B, PreHevbrio, and Recombivax-HB) may be completed on a routine 6-month schedule or an accelerated 16-week schedule if using minimum dosing intervals [12]. Patients receiving a three-dose product on a minimum interval schedule achieved vaccine protection levels similar to those who were vaccinated over six or more months [11]. EHR prompts and other tools should ensure that providers and patients are informed of this accelerated option.

## 5. Summary of HBV Screening Recommendations

In March 2023, the CDC updated recommendations for hepatitis B screening in all adults aged 18 years or older [6]. They now recommend universal HBV screening of all adults using a one-time, triple-test serologic panel (triple panel) that includes hepatitis B surface antigen (HBsAg), hepatitis B surface antibody (anti-HBs, HBsAb), and core (total) antibody (anti-HBc total, HBcAb total). This testing would identify those with chronic infection, those who had a past infection and are at risk of reactivation with immunosuppression, and those who may benefit from vaccination. Pregnant individuals should continue to be tested for HBsAg during each pregnancy as part of their prenatal care, regardless of their testing or vaccination history. CDC also recommends that susceptible individuals who are not vaccinated after screening and who are at high risk of infection should be tested periodically. Additionally, anyone who requests HBV testing should also be tested [6]. Note that HBV screening is not a prerequisite to vaccination, but if both are occurring, it is important to ensure HBV screening data are factored into HBV vaccination prompts.

## 6. EHR Implementation for Screening

As with vaccination, building HBV screening in an EHR consists of three components: patient identification and triggers, order sets, and documentation and patient follow-up.

### 6.1. Patient Identification and Triggers

Identify patients who meet the age eligibility criteria (age 18 years or older) who do not have evidence of a prior hepatitis B infection (not already known to be positive for HBsAg or anti-HBc) and who have not previously been screened with a triple panel.

### 6.2. Order Sets

- Bundle the ordering of all three recommended HBV serologic tests into one screening panel to prevent ordering errors.
- Include, when possible, the rationale for testing and evidence-based references on demand.

### 6.3. Documentation and Patient Follow-Up

- Chart documentation (what was ordered and why):
  ▪ Consider the policy of screening and vaccination at the same visit.
- If possible, include guidance for results interpretation (Table 3):
  ▪ Repeat testing for isolated HBsAg positive cases since this is often a false positive;
  ▪ Documentation on the problem list of “risk of HBV reactivation with immunosuppression” for all patients with positive core antibody total (anti-HBc total) results.
- Construct templates or smart phrases for patient communication (via electronic patient portal, written, or verbal communication) for each testing outcome.
- Schedule follow-up visits and additional orders, as necessary.

### 6.4. Special Considerations

Screen and administer first HBV vaccine dose on the same day, as recommended by the CDC [6]. When feasible, phlebotomy for screening should occur before vaccine administration because HBV vaccination can lead to transiently false positive HBsAg test results for several days following vaccination. If the vaccine is administered first, the CDC recommends waiting at least 30 days before phlebotomy for HBV screening [13].

## 7. HBV Screening Results

Table 3 illustrates the possible serologic profiles that can result from the three-panel HBV screening test (HBsAg, anti-HBs, anti-HBc total) and briefly reviews the recommended next steps associated with these results. Other hepatitis B tests, such as the hepatitis B core IgM antibody test, hepatitis B e-antigen (HBeAg), or HBV viral level (HBV DNA quantitation), are considered secondary tests reserved primarily for those who are actively infected or at risk of HBV reactivation to help distinguish and further characterize these individuals.

## 8. Frequently Asked Questions

Here we present several questions that are likely to arise in the course of designing EHR support for HBV screening or vaccination.

### 8.1. What Are the Goals of Electronic Medical Decision Support for Hepatitis B Vaccination with or without HBV Screening?

EHR support (including prompts, task lists, or order sets) described in this document are population-level recommendations that reflect public health guidelines and facilitate best practices. The accurate selection of patients for testing or vaccination and decision support through order sets can reduce cognitive burden and time demands on providers. Special populations may have alternative workstreams and recommendations that would be addressed via population-specific support (e.g., people receiving dialysis, immunocompromised patients, or people with ongoing risk of HBV acquisition, such as those who use drugs or those who work in direct patient care settings at risk of percutaneous injury).

### 8.2. Who Should Receive the EHR Prompts or Reminders for HBV Vaccination?

The audience for these prompts or reminders should be considered broadly to extend beyond clinicians who see the patients and should include pharmacists, medical assistants, and nurses. Anyone who is capable of administering a vaccine dose can be prompted to ensure completion of the series.

### 8.3. Can a Health System Conduct a Hepatitis B Vaccination Program without Implementing an HBV Screening Program?

Yes. In this case, individuals aged 19–59 years who do not have documentation of completion of HBV vaccination will generate a prompt for hepatitis B vaccination. There is a risk that a person may have unrecognized chronic HBV infection before vaccination and be unaware of the potential clinical consequences or need for treatment. Vaccination would have no effect on pre-existing HBV infection. It is therefore recommended that the need for triple-panel screening be flagged (in task list or other format) if such testing is not in the system.

### 8.4. Can a Health System Conduct an HBV Screening Program without Implementing a Hepatitis B Vaccination Program?

Yes, but it is recommended that a collaboration is established so anyone who is identified as nonimmune is referred for vaccination and that the need for hepatitis B vaccination be added to the task list.

### 8.5. What Are Options for the Timing of HBV Screening and Vaccination Initiatives?

Hepatitis B screening and vaccination can occur on the same visit for individuals who meet criteria for both prompts. If someone’s HBV status is unknown, systems may choose to initiate the hepatitis B vaccine series and interrupt the series if they are found to have prior exposure to HBV (positive anti-HBc with or without HBsAg) or immune protection (isolated anti-HBs). Ideally, phlebotomy for screening tests should be performed before the HBV vaccine is administered to avoid the risk of a false positive HBsAg result. If phlebotomy services are not co-localized with clinics, this may be logistically difficult. Another option is to place a pre-visit order for the HBV triple-panel screening test. This strategy will also allow the clinician to use the screening data in decision making regarding the need for vaccination.

### 8.6. Are Hepatitis B Vaccines Covered by Health Insurance Plans in the US?

Yes. The Affordable Care Act (ACA) requires commercial and employee-sponsored plans to cover all ACIP-recommended vaccines administered by “in-network” providers, and most plans will cover with no cost-sharing to the patient. The Inflation Reduction Act of 2022 mandated that all ACIP-recommended vaccines be covered with no cost-sharing to the patient by Medicaid and Medicare (hepatitis B is covered by Part B for people at intermediate or high risk of hepatitis B and covered by Part D for people at low risk) [15,16]. For individuals without insurance, some public health clinics may provide HBV vaccination without cost.

### 8.7. How Should a Positive HBsAg Result (with Anti-HBc Total also Positive and Anti-HBs Negative) Be Handled?

Most individuals with positive HBsAg and positive anti-HBc total results have chronic (rather than acute) infection. The definition of chronic infection requires two positive HBsAg tests at least six months apart. For patients with positive HBsAg and anti-HBc total, it is recommended that “active HBV infection” be added to the problem list (which includes both acute and chronic HBV infections). Some labs perform reflex quantitative HBV DNA testing from the same sample since this result helps determine the need for antiviral therapy.

### 8.8. How Should an Isolated Anti-HBc Total Antibody Result (Negative Results for HBsAg and Anti-HBs Antibody) Be Addressed?

It is recommended to add a note to the problem list indicating “risk of HBV reactivation with immunosuppression”. These patients may benefit from consultation with someone familiar with hepatitis B infection.

### 8.9. How Should Anti-HBs Positive, Anti-HBc Total Positive, and HBsAg Negative Results Be Handled?

These individuals have prior exposure to HBV infection and have evidence of natural immunity; thus, they do not need HBV vaccination. It is recommended to add a note to the problem list indicating “risk of HBV reactivation with immunosuppression”.

### 8.10. How Should Isolated HBsAg Results (Negative Anti-HBs and Anti-HBc Total Results) Be Handled?

This profile most likely represents a false positive result. Possible explanations include (1) drawing blood for HBV screening within 30 days of HBV vaccination (including drawing blood for testing immediately after HBV vaccination) since the HBsAg particles that comprise the HBV vaccine are detected by HBsAg assays or (2) contamination of the blood sample by a previous sample that contained HBsAg [14,17]. Add a task list recommendation to repeat the HBV screening panel in 30 days [13].

### 8.11. Do Individuals Born in the US during Universal Hepatitis B Vaccination (either Infant or Adolescent Catch-Up Programs) Need to Be Vaccinated if Their HBV Screening Panels Do Not Show Immunity to HBV (Negative Anti-HBs)?

If individuals have documentation of completing their childhood hepatitis B vaccination series, they do not need a prompt for vaccination, even if anti-HBs results are negative. Systems may choose to initiate the hepatitis B vaccine series and interrupt the series if documentation is provided and entered into EHR. Although routine childhood hepatitis B vaccination rates among people born in the United States since 1991 are high, the CDC’s general best practice recommendation is to offer vaccination in the absence of documented vaccination [18].

### 8.12. If a Health System Is Not Recommending Hepatitis B Vaccination for Individuals Aged 60 Years or Older at Low Risk of HBV Infection, Does the Triple-Panel HBV Screening Panel Need to Be Performed?

The CDC recommends triple-panel HBV screening for all adults, even in settings where HBV vaccination is not offered to the patient at the time by the health system or a healthcare provider [6].

## 9. Conclusions

Implementation of HBV vaccination prompts in EHR can be complicated, especially if a healthcare system is also implementing HBV screening prompts, due to questions around eligibility, HBV status, timing, vaccine product choice, and workflow. This guide is intended to help clinical champions, EHR review committees, laboratory medicine, and information technology professionals develop medical support strategies that are easy to follow and target the appropriate patients. As health systems work towards the implementation of the new CDC recommendations, it will be important to document and share key learnings and strategies on the technical implementation of EHR schematics, the adapting of EHR tools for key subgroups, and the barriers to implementation. Scalable strategies that leverage the EHR and facilitate HBV screening and vaccination will ultimately bring us closer to the goal of HBV elimination.

## Figures and Tables

**Table 1:** Adults aged ≥60 years with risk factors for hepatitis B acquisition and recommendations for vaccination [5,6].

Persons at risk of infection from sexual exposure: ○ Sex partners of persons testing positive for HBsAg; ○ Sexually active persons who are not in a long-term, mutually monogamous relationship (e.g., persons with more than one sex partner during the previous 6 months); ○ Persons seeking evaluation or treatment for a sexually transmitted infection; ○ Men who have sex with men. Persons at risk of infection from percutaneous or mucosal exposure to blood: ○ Persons with current or recent drug use via injection; ○ Household contacts of persons testing positive for HBsAg; ○ Residents and staff members of facilities for persons with developmental disabilities; ○ Health care and public safety personnel with reasonably anticipated risk of exposure to blood or blood-contaminated body fluids; ○ Persons on maintenance dialysis, including in center or home hemodialysis and peritoneal dialysis, and persons who are predialysis; ○ Persons with diabetes, at the discretion of the treating clinician. Others: ○ International travelers to countries with high or intermediate levels of endemic hepatitis B virus infection (HBsAg prevalence of ≥2%, https://www.ncbi.nlm.nih.gov/pmc/articles/PMC9997714/#B3 (accessed on 16 November 2023)); ○ Persons with hepatitis C virus infection; ○ Persons with chronic liver disease (including, but not limited to, persons with cirrhosis, fatty liver disease, alcoholic liver disease, autoimmune hepatitis, and an alanine aminotransferase or aspartate aminotransferase level greater than twice the upper limit of normal); ○ Persons with HIV infection; ○ Persons who are incarcerated. Adults aged ≥60 years without known risk factors for hepatitis B may receive hepatitis B vaccines.

**Table 2:** Currently available monovalent hepatitis B vaccines in the United States [5,11].

Age Groups for Vaccines	Routine Schedules	Special Populations
Engerix-B—HepB Ages: all ages	Three doses 0, 1 month, and 4–6 months	Pregnant patients: recommended Dialysis patients: four-dose series at 0, 1, 2, and 6 months (note: use 2 mL dose instead of the normal adult dose of 1 mL)
Heplisav-B—HepB Ages: 18 years and older	Two doses 0 and 1 month	Pregnant patients: not recommended due to lack of safety data Dialysis patients: not recommended due to lack of efficacy data
PreHevbrio—HepB Ages: 18 years and older	Three doses 0, 1, and 4–6 months	Pregnant patients: not recommended due to lack of safety data Dialysis patients: not recommended due to lack of efficacy data
Recombivax HB—HepB Ages: all ages Dialysis formulation is for 18 years and older	Three doses 0, 1 month, and 4–6 months	Pregnant patients: recommended Dialysis patients: recommended, at 0, 1, and 6 months (note: use dialysis formulation 1 mL = 40 mcg)

For details, see the current CDC-recommended adult immunization schedule (https://www.cdc.gov/vaccines/schedules/hcp/imz/adult.html (accessed on 6 February 2024)).

**Table 3:** Hepatitis B serologic profiles from the three-test panel [13,14].

Profile	HBsAg	Anti-HBc Total	Anti-HBs	Action/Comments
Never infected (susceptible)	-	-	-	Needs HBV vaccination. Individuals with a remote history of vaccination may show undetectable anti-HBs. In the absence of specific indications for revaccination, no vaccination is recommended for a person with a negative anti-HBs and documentation of complete vaccination.
Acute infection	+	+	-	Clinical history and presence of significant ALT/AST elevation (>5–10 times upper limit of normal) can generally distinguish acute from chronic infection. These patients are often anti-HBc IgM positive.
Immune control (past infection)	-	+	+	Considered to have natural immunity. May warrant further management if undergoing certain immunosuppressive therapy due to risk of HBV reactivation with these treatments. No action needed.
Chronic infection	+	+	-	Can obtain HBV DNA quantitation if not already obtained from reflex testing. Consider referral for further HBV care.
Isolated core antibody	-	+	-	Most of these individuals represent those with natural immunity in whom anti-HBs have waned. May warrant further management if infected with HIV or if undergoing hepatitis C or immunosuppressive therapy due to risk of HBV reactivation with these treatments.
Immunized (or passive Ab receipt)	-	-	+	Nothing to do if immunized.

## References

[1] World Health Organization (2017). Global Hepatitis Report 2017. https://www.who.int/publications/i/item/9789241565455.

[2] Kim H.S., Rotundo L., Yang J.D., Kim D., Kothari N., Feurdean M., Ruhl C., Unalp-Arida A. (2017). Racial/ethnic disparities in the prevalence and awareness of Hepatitis B virus infection and immunity in the United States. J. Viral Hepat..

[3] Coalition for Global Hepatitis Elimination (2023). USA National Hepatitis Elimination Profile. Coalition for Global Hepatitis Elimination. https://www.globalhep.org/sites/default/files/content/national_profiles/files/2024-01/National%20Hepatitis%20Elimination%20Profile%20-USA-2023%20update-Oct23.pdf.

[4] Lu P.-J., Hung M.-C., Srivastav A., Grohskopf L.A., Kobayashi M., Harris A.M., Dooling K.L., Markowitz L.E., Rodriguez-Lainz A., Williams W.W. (2021). Surveillance of Vaccination Coverage Among Adult Populations—United States, 2018. MMWR Surveill. Summ..

[5] Weng M.K., Doshani M., Khan M.A., Frey S., Ault K., Moore K.L., Hall E.W., Morgan R.L., Campos-Outcalt D., Wester C. (2022). Universal Hepatitis B Vaccination in Adults Aged 19–59 Years: Updated Recommendations of the Advisory Committee on Immunization Practices—United States, 2022. MMWR Morb. Mortal. Wkly. Rep..

[6] Conners E.E., Panagiotakopoulos L., Hofmeister M.G., Spradling P.R., Hagan L.M., Harris A.M., Rogers-Brown J.S., Wester C., Nelson N.P., Rapposelli K. (2023). Screening and Testing for Hepatitis B Virus Infection: CDC Recommendations—United States, 2023. MMWR Recomm. Rep..

[7] Raban M.Z., Gates P.J., Gamboa S., Gonzalez G., Westbrook J.I. (2023). Effectiveness of non-interruptive nudge interventions in electronic health records to improve the delivery of care in hospitals: A systematic review. J. Am. Med. Inform. Assoc..

[8] Weir R.C., Toyoji M., McKee M., Li V., Wang C.C. (2018). Assessing the Impact of Electronic Health Record Interventions on Hepatitis B Screening and Vaccination. J. Health Care Poor Underserved.

[9] Patel M.S., Volpp K.G., Small D.S., Wynne C., Zhu J., Yang L., Honeywell S., Day S.C. (2017). Using Active Choice Within the Electronic Health Record to Increase Influenza Vaccination Rates. J. Gen. Intern. Med..

[10] Patel M.S., Volpp K.G., Asch D.A. (2018). Nudge Units to Improve the Delivery of Health Care. N. Engl. J. Med..

[11] Schillie S., Vellozzi C., Reingold A., Harris A., Haber P., Ward J.W., Nelson N.P. (2018). Prevention of Hepatitis B Virus Infection in the United States: Recommendations of the Advisory Committee on Immunization Practices. MMWR Recomm. Rep..

[12] Centers for Disease Control and Prevention Recommended Adult Immunization Schedule for Ages 19 Years or Older 2024. Centers for Disease Control and Prevention. 2023, Volume 5. https://www.cdc.gov/vaccines/schedules/downloads/adult/adult-combined-schedule.pdf.

[13] Centers for Disease Control and Prevention Interpretation of Hepatitis B Serologic Test Results. CDC. www.cdc.gov. Published 3 March 2023. http://www.cdc.gov/hepatitis/hbv/interpretationOfHepBSerologicResults.htm.

[14] Cororran M., Spach D., Kim H.N. Core Concepts—HBV Screening, Testing, and Diagnosis—Screening and Diagnosis—Hepatitis B Online. www.hepatitisb.uw.edu. Published 13 March 2023. http://www.hepatitisb.uw.edu/go/screening-diagnosis/diagnosis-hbv/core-concept/all.

[15] Yarmuth J.A. (2022). Inflation Reduction Act of 2022. https://www.congress.gov/bill/117th-congress/house-bill/5376.

[16] National Adult and Influenza Immunization Summit (2023). Insurance Coverage of Adult Immunizations. National Adult and Influenza Immunization Summit. https://www.izsummitpartners.org/content/uploads/NAIIS_vaccine-insurance-coverage_2023.pdf.

[17] National Adult and Influenza Immunization Summit (2023). Implementing Hepatitis B Universal Adult Screening and Vaccination: Clinical Answers for Healthcare Professionals. National Adult and Influenza Immunization Summit. https://www.immunize.org/wp-content/uploads/catg.d/p2080.pdf.

[18] Centers for Disease Control and Prevention Timing and Spacing of Immunobiologics: General Best Practice Guidelines for Immunization. www.cdc.gov. Published 19 September 2023. https://www.cdc.gov/vaccines/hcp/acip-recs/general-recs/timing.html#unknown.

